# Characteristics and patterns of older adult homicides in the United States

**DOI:** 10.1186/s40621-021-00299-w

**Published:** 2021-02-01

**Authors:** Riffat Ara Shawon, Avanti Adhia, Christopher DeCou, Ali Rowhani-Rahbar

**Affiliations:** 1 Department of Epidemiology, School of Public Health, University of Washington, Seattle, Washington USA; 2 Firearm Injury & Policy Research Program, Harborview Injury Prevention & Research Center, University of Washington, Seattle, Washington USA; 3 Department of Pediatrics, School of Medicine, University of Washington, Seattle, Washington USA; 4 Department of Psychiatry and Behavioral Sciences, School of Medicine, University of Washington, Seattle, Washington USA

**Keywords:** Older adults, Homicide, Homicide-suicide, Multiple victims, Intimate partner, Violence, Firearms, National Violent Death Reporting System (NVDRS)

## Abstract

**Background:**

Nearly 1800 homicides were reported in 2018 among individuals aged 60 years or older in the US. The characteristics and circumstances of these homicides are understudied. We investigated the trends of homicides over time and compared victim, perpetrator, and incident characteristics by mechanism (firearm vs. non-firearm) among adults aged 60 years or older.

**Methods:**

We examined cross-sectional restricted-access data from the National Violent Death Reporting System from 2003 to 2017. Fatal Injury Data by CDC provided age-adjusted homicide rates. We quantitatively summarized victim, perpetrator, and incident characteristics for firearm and non-firearm homicides. We also reviewed 150 qualitative narratives to better understand the context of older adult homicides perpetrated by firearms. All data were analyzed in February 2020.

**Results:**

Overall and firearm-specific older adult homicide rates increased between 2014 and 2017. Of the 6188 victims, 62% were male. The majority of victims (68%) were killed at home. Firearms (44%), sharp (19%) and blunt weapons (15%) were common mechanisms used in older adult homicides. The perpetrator was an intimate partner in 39% of firearm homicides and 12% of non-firearm homicides (prevalence difference = 27%; 95% CI: 25, 30%). Similarly, homicide-suicides (prevalence difference = 21%; 95% CI: 19, 22%) and multiple-victim incidents (prevalence difference = 7%; 95% CI: 5, 8%) were more common in firearm (23 and 13%, respectively) than in non-firearm (2 and 6%, respectively) homicides. Common contexts of firearm homicides were familial/intimate partner problems, robbery/burglary, argument, and illness.

**Conclusions:**

A substantial number of older adults were killed with firearms and by their intimate partners. Further research to identify violence victimization prevention strategies in this group, especially those that limit access to firearms by potential perpetrators, is warranted.

**Supplementary Information:**

The online version contains supplementary material available at 10.1186/s40621-021-00299-w.

## Background

Violence is a major cause of disability and death among individuals aged 60 years or older (hereafter: older adults) in the US (Stevens et al. 1999). Nearly 1800 older adults died by homicide in 2018 (Centers for Disease Control and Prevention 2019). Recent trends indicate that the rates of older adult homicides are increasing, particularly for males (Logan et al. 2019). Older adult homicides constituted 9.3% of the total homicides in 2018 compared to 6.5% in 2001 (Centers for Disease Control and Prevention 2019). Homicide among older adults contributes to a significant number of lives lost and lower quality of life among members of this age group due to a heightened sense of vulnerability and fear (Nelsen and Huff-Corzine 1998). The older adult population, as a proportion of the total population, is increasing at an unprecedented rate in the US. With the current demographic shifts, it is predicted that 28% of the total population will be older adults by 2050 (United Nations, Department of Economic and Social Affairs PD 2017). Hence, the impact of violence in this age group will likely become larger in the future.

Homicide victimization predominately affects the younger population (Bachman and Meloy 2008). As a consequence, older adult homicide is under-researched. A systematic review of the literature between 1982 and 2018 highlighted characteristics of older adult homicides from 33 studies. According to the findings, approximately one-half of the victims were female, while perpetrators were predominantly male and younger than the victim (Rogers and Storey 2019). In most instances, the perpetrator was known to the victim such as a family member, an intimate partner, a friend, or an acquaintance (Rogers and Storey 2019). Declining health and cognition, low mobility, social isolation, and a history of abuse were the primary risk factors for homicide victimization (Rogers and Storey 2019). The systematic review reported that homicide mechanisms varied by perpetrator’s physical and mental health conditions. Relief of caregiver burden, monetary benefit, revenge, sexual abuse, self-defense, and mercy killing were some of the noted motives for the murders. Older adults, women in particular, were mostly killed in their homes (Rogers and Storey 2019). ﻿Firearms were by far the most common (42%) mechanism used in older adult homicides (Logan et al. 2019).

Literature on older adult homicide is often limited in scope (e.g., including a small number of cities, counties, or states) or based on data collected over 10 years ago. Previous studies have compared homicide incidents between older adults and younger victims (Abrams et al. 2007) or between males and females (Krienert and Walsh 2010) but were limited in describing detailed characteristics of perpetrators and incidents. To our knowledge, no study has yet examined differences in older adult homicides by injury mechanism across multiple states in the US while also including an assessment of the narratives describing the circumstances of those deaths.

We sought to address these knowledge gaps by using a national data set of older adult homicides to examine detailed victim, perpetrator, and incident characteristics. Since firearms is the most common mechanism for older adult homicide, we were interested in comparing homicides that involved firearms with those that did not involve firearms. We further aimed to characterize the context and circumstances of firearm homicides using qualitative narrative data. This study contributes contemporary and actionable information to the extant body of literature on older adult homicides and informs prevention-focused policy, programs, and practices that can address this important public health problem.

## Methods

We used the most recent available data from the National Violent Death Reporting System (NVDRS) Restricted Access Database to compare victim, perpetrator, and incident characteristics by homicide mechanism (firearm vs. non-firearm) among older adults, and Fatal Injury Data from Web-based Injury Statistics Query and Reporting System (WISQARS) of the Centers for Disease Control and Prevention (CDC) to examine trends of these homicides over time. We defined ages 60 years or older as “older adults” as per the Uniform Definitions and Recommended Core Data Elements by CDC (Hall et al. 2016). Institutional Review Board approval was not required for the use of secondary de-identified data used in this study.

### Data source

NVDRS is a state-based active surveillance system for violent deaths including homicides, suicides, and unintentional firearm deaths. State-level data abstractors link data from vital records, coroner or medical examiner reports, and law enforcement reports and code detailed information using CDC-developed guidelines (National Center for Injury Prevention and Control 2018). The abstractors also prepare narrative summaries from medical/coroner and law enforcement reports with incident-level details that contain information about circumstances that principiated the violent death gathered through interviews with the victim’s friends and family, suicide notes, toxicology reports, and other available information. This unique attribute of data linkage and relatively more complete information on the victim-offender relationship and homicide circumstances is a strength of NVDRS over other alternatives such as the Supplementary Homicide Reports (SHR) by the Federal Bureau of Investigation (FBI). The scope of underreporting and missing data on homicides is also smaller in NVDRS compared to SHR (Shields and Ward 2008).

For certain variables, like perpetrator information, the amount of missingness was not negligible in our NVDRS data. We have indicated the proportion of missing data for each variable in the table footnotes so that readers can exercise caution interpreting our results for certain variables that have high magnitude of missingness. We also examined whether the extent of missingness was differential based on the main categories of interest (i.e., firearm vs. non-firearm). Supplemental Table S3 indicate no notable differences between data missingness in firearm and non-firearm homicide groups.

There were no missing data for binary variables (“yes” or “no”) on incident characteristics. Nonetheless, according to NVDRS documentations (National Center for Injury Prevention and Control 2018), the response “no” in case of incident characteristic in particular could either mean absence of the circumstance or lack of confirmation of its presence. We had access to narrative summaries from medical/coroner reports and the law enforcement reports for each homicide case. For some cases, either medical/coroner reports or law enforcement or both narratives were missing. Some narratives were more detailed than the others depending on the information available to the abstractor from the medical/coroner and law enforcement reports at the time of abstraction. Both the medical/coroner and law enforcement narrative summaries included information about the context of the homicide when the information was known.

We used abstractor coded data for all older adult homicides from 2003 to 2017 for 36 available states (Alaska, Arizona, California, Colorado, Connecticut, Delaware, Georgia, Hawaii, Illinois, Indiana, Iowa, Kansas, Kentucky, Maine, Maryland, Massachusetts, Michigan, Minnesota, Nevada, New Hampshire, New Jersey, New Mexico, New York, North Carolina, Ohio, Oklahoma, Oregon, Pennsylvania, Rhode Island, South Carolina, Utah, Vermont, Virginia, Washington, West Virginia, and Wisconsin), Puerto Rico, and District of Columbia. Statewide data availability varied by year as states were progressively added into the system. To assess the representativeness of our data for the total US population, we compared the victim characteristics from our study with those from WISQARS since WISQARS has data available for all 50 states and the District of Columbia. The results were fairly comparable between the two data sources in terms of the distribution of selected characteristics (Supplemental Table S1). Fatal Injury Data from WISQARS is a source of counts and age-adjusted rates of injury mortality by mechanism and manner of death based on the *International Classification of Diseases* - 10th revision.

We reviewed all of the qualitative narrative summaries available for a random sample of 150 older adult homicide cases to better understand the context of older adult homicides perpetrated using firearms. To obtain the sample, we first excluded the cases where both medical/coroner and law enforcement narrative summaries were missing. We generated random numbers such that each case had a random number associated with them in the data. With ascending sorting, we kept the first 150 smallest random numbers and thus obtained 150 random cases with narrative data. In the resulting sample, law enforcement narrative summary was missing for 10 cases and medical/coroner narrative summary was missing for 2 cases. For the remaining 138 cases, both types of narrative summaries were available.

### Measures

We first described homicide mechanisms based on mechanisms used for homicide including firearms, sharp instruments, blunt instruments, hanging/strangulation/suffocation, personal weapon (e.g., hands, feet, fists) or other (e.g., poisoning, fall, fire/burn, electrocution, explosives, non-powder guns, nail gun, taser, shaking, motor vehicle and other transport vehicles, intentional neglect, biological weapon, exposure to weather conditions). To compare victim, perpetrator, and incident characteristics by mechanisms, we dichotomized the injury mechanism into firearm vs. non-firearm. We reported perpetrator information for the person identified in NVDRS as the primary suspect in the incident. We dichotomized victim’s education level as obtaining less than a high school degree vs. a high school degree or above. We categorized race and ethnicity as White, non-Hispanic, Black, non-Hispanic, Hispanic, and all other races (Adhia et al. 2019). Victim-perpetrator relationship was coded as intimate partners, family members (other than the spouse), acquaintances (e.g., friends, colleagues), and strangers. NVDRS defines intimate partner as a current or ex-partner including boyfriend, girlfriend, dating partner, sexual partner, or spouse. We categorized location of injury as victim’s home, other homes/apartments, street/sidewalk or alley, parking lot/garage or motor vehicle, and other locations. When at least one additional death occurred (either a suicide or homicide) as part of the incident, we classified it as a multiple-victim incident.

NVDRS also contains several incident circumstances (e.g., homicide-suicide, intimate partner violence-related, mercy killing, justifiable self-defense, alcohol use, drug involvement, gang-related) coded as yes/present or no/not present/unknown. Complete definitions for all incident circumstances variables used in this study are provided in the appendix (Supplemental Table S2).

### Statistical analysis

To investigate firearm vs. non-firearm homicides, we excluded 251 (3.9%) of the initial 6439 older adult homicides where the mechanism was unknown. A total of 5961 homicide incidents with 6188 older adult victims were included in this analysis. Homicide incident and victim numbers were different as some of the incidents included more than one victim. Age-adjusted rates were generated for older adult homicides overall and separately for firearms and non-firearm incidents using WISQARS. We used descriptive statistics to characterize firearm and non-firearm homicides in terms of victim, perpetrator, and incident features. We calculated prevalence differences and constructed their corresponding 95% confidence intervals using the exact method based on binomial distribution. STATA version 12 (StataCorp 2011) was used for all analyses.

To understand the contexts of firearm homicides among older adults using narrative data, we began with a list of common contexts identified from the circumstance variables in the quantitative analysis and from prior literature (Rogers and Storey 2019). This list expanded as themes emerged through iterative review of the records. Authors RAS and ARR examined and categorized the narratives and reached a consensus on predominant contexts. This process yielded four major themes. Themes with a smaller number of cases were grouped into the “other” category. We present results with examples created by combining information from multiple narratives with some alterations to avoid identification of victim/perpetrator identity per CDC recommendations.

## Results

**Fig. 1:**
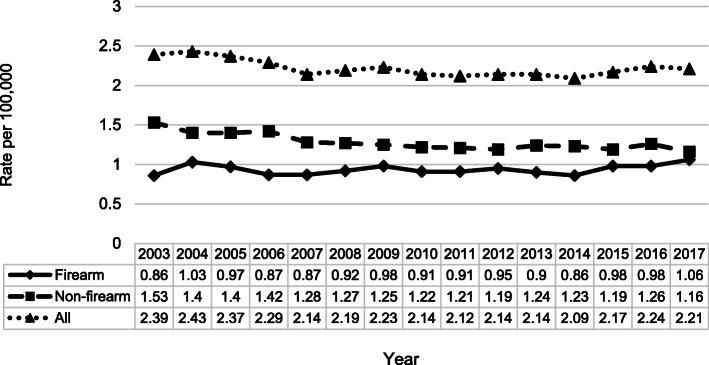
Age-adjusted older adult homicide rates in the U.S. from WISQARS Fatal Injury Data

Firearm and non-firearm homicide rates in older adults were fairly consistent between 2003 and 2013 with a modest but steady increase in firearm homicides since 2014 (Fig. 1). Firearms were the most common mechanism used (44%), followed by sharp instruments (19%), blunt instruments (15%), personal weapons (9%), hanging and strangulation (6%), and other (6%). When the type of firearm used was known, 78% of firearm homicides were perpetrated by handguns, 13% by shotguns, and 9% by rifles.

The majority of the firearm victims were male (63%), non-Hispanic White (66%) with a mean age of 69 years (Table 1). Non-firearm victims were 62% male and 64% White non-Hispanic and had a mean age of 71 years. About 88% of both firearm and non-firearm homicides were perpetrated by someone close to the victim in relationship or someone the victim knew. About 51% of firearm victims were married or in a civil union/domestic partnership compared to 27% of non-firearm victims. The largest proportion (39%) of firearm victims were killed by their current or ex-intimate partners, whereas the fraction was comparatively lower (12%) among non-firearm victims (prevalence difference = 27%; 95% CI: 25, 30%). Victims were killed in their own homes in the majority of firearm (66%) and non-firearm (71%) homicides. (Table 1).

**Table 1:** Distribution of older adult homicide victim characteristics using the National Violent Death Reporting System Data from 2003 to 2017

Victim Characteristics^a^	Total (*N* = 6188) *N (%)*	Firearm Homicides (*n* = 2710) *n (%)*	Non-firearm Homicides (*n* = 3478) *n (%)*	Prevalence Difference (95% CI)
Sex				
*Male*	3859 (62.4)	1704 (62.9)	2155 (62.0)	0.9 (−1.5, 3.3)
*Female*	2329 (37.6)	1006 (37.1)	1323 (38.0)	−0.9 (−3.3, 1.5)
Race/Ethnicity				
*White, non-Hispanic*	4018 (64.9)	1795 (66.2)	2223 (63.9)	2.3 (−0.1, 4.7)
*Black, non-Hispanic*	1499 (24.2)	668 (24.7)	831 (23.9)	0.8 (−1.4, 2.9)
*Hispanic*	351 (5.6)	122 (4.5)	229 (6.6)	−2.1 (−3.2, −0.9)
*All other race*^*b*^	320 (5.2)	125 (4.6)	195(5.6)	−0.9 (−2.1, 0.1)
Age (years)				
*60 to 64*	2123 (34.3)	1026 (37.9)	1097 (31.5)	6.3 (3.9, 8.7)
*65 to 74*	2349 (38.0)	1099 (40.6)	1250 (35.9)	4.6 (2.2, 7.1)
*75 to 84*	1216 (19.7)	459 (16.9)	757 (21.8)	−4.8 (−6.8, −2.9)
*85 and above*	500 (8.1)	126 (4.7)	374 (10.8)	−6.1 (−7.4, −4.8)
Education level				
*Less than high school degree*	1471 (26.4)	574 (23.3)	897 (28.8)	−5.5 (−7.8, −3.2)
*High school degree or above*	4111 (73.7)	1891 (76.7)	2220 (71.2)	5.5 (3.2, 7.8)
Marital status				
*Married/civil union/domestic partnership*	2276 (37.7)	1351 (50.7)	925 (27.4)	23.3 (20.8, 25.7)
*Divorced/separated/widowed/never married/single*	3767 (62.3)	1316 (49.3)	2451 (72.6)	−23.3 (−25.7, −20.8)
Victim’s relationship with perpetrator				
*Family member (other than spouse)*	1283 (32.9)	453 (27.1)	830 (37.3)	−10.3 (−13.2, −7.3)
*Intimate partner (current or ex)*	916 (23.5)	655 (39.1)	261 (11.7)	27.4 (24.7, 30.1)
*Acquaintances (includes friends, colleagues* etc.*)*	1249 (32.0)	374 (22.3)	875 (39.3)	−17.0 (−19.8, − 14.2)
*Stranger*	450 (11.5)	192 (11.5)	258 (11.6)	−0.1 (−2.2, 1.9)
Victim’s location when injured				
*Victim’s home*	4134 (68.4)	1728 (65.8)	2406 (70.5)	−4.7 (−7.1, − 2.3)
*Other home, apartment*	499 (8.3)	249 (9.5)	250 (7.3)	2.2 (0.7, 3.6)
*Street, sidewalk, alley*	417 (6.9)	191 (7.3)	226 (6.6)	0.7 (−0.6, 1.9)
*Motor vehicle, parking lot/garage*	209 (3.5)	129 (4.9)	80 (2.3)	2.6 (1.6, 3.5)
*Other*	782 (12.9)	330 (12.6)	452 (13.2)	−0.7 (−2.4, 1.0)
Served in military	1366 (24.0)	576 (22.8)	790 (25.0)	−2.3 (−4.5, −0.03)
Homeless	93 (1.6)	14 (0.5)	79 (2.4)	−1.8 (−2.4, − 1.2)
Alcohol use suspected when injured	660 (13.4)	247 (11.7)	413 (14.7)	−3.1 (15.0, −1.2)
Alcohol problem	237 (3.8)	80 (3.0)	157 (4.5)	−1.6 (−2.5, −0.6)
Other substance problem	193 (3.1)	74 (2.7)	119 (3.4)	−0.7 (−1.6, 0.2)
Current diagnosed mental health problem	405 (6.5)	152 (5.6)	253 (7.3)	−1.7 (−2.9, −0.4)
Current depressed mood	62 (1.0)	33 (1.2)	29 (0.8)	0.4 (−0.1, 0.9)
Current mental health or substance abuse treatment	211 (3.4)	84 (3.1)	127 (3.7)	−0.6 (−1.5, 0.4)
Ever treated for mental health or substance abuse	263 (4.3)	100 (3.7)	163 (4.7)	−1.0 (− 1.2, 0.002)
Victim was a bystander	86 (1.4)	59 (2.2)	27 (0.8)	1.4 (0.7, 2.0)
Victim was intervener assisting another victim	44 (0.7)	14 (0.5)	30 (0.9)	−0.4 (−0.8, 0.06)
Victim used weapon	156 (2.5)	100 (3.7)	56 (1.6)	2.1 (1.3, 2.9)
Prior history of victim abuse by perpetrator	171 (4.1)	54 (2.9)	117 (5.0)	−2.1 (−3.3, −0.9)
Victim was perpetrator of violence in past month	40 (0.7)	22 (0.8)	18 (0.5)	0.3 (−0.2, 0.7)
Victim experienced violence in past month	87 (1.4)	29 (1.1)	58 (1.7)	−0.6 (−1.2, − 0.02)

Columns may not always equal to 100% due to rounding

^a^ Missing Data: Education level (606 [9.8%]), Marital status (145 [2.3%]), Victim’s relationship with perpetrator (2290 [37.0%]), Victim’s location when injured (147 [2.4%]), Served in military (497 [8.0%]), Homeless (236 [3.8%]), Alcohol use suspected when injured (1269 [20.5%]), Prior history of victim abuse by perpetrator (1994 [32.2%])

^b^ all other race includes American Indian/Alaska Native (non-Hispanic), Asian/Pacific Islander (non-Hispanic), two or more races (non-Hispanic), other/unspecified (non-Hispanic)

The majority of perpetrators were male in both firearm (89%) and non-firearm homicides (84%) (Table 2). On average, perpetrators who used firearms were older (mean age 50 years) than those who used other mechanisms (mean age 41 years). The proportion of perpetrators identified as having a mental illness was substantially lower among those who used firearms (9%) compared to those who used other mechanisms (18%). About 4% of firearm and 6% of non-firearm homicide perpetrators were caregivers of the older adults who could be family members, intimate partners, or external caregivers.

**Table 2:** Distribution of older adult homicide perpetrator characteristics using the National Violent Death Reporting System Data from 2003 to 2017

Perpetrator Characteristics^a^	Total (*N* = 5961) *N (%)*	Firearm Homicides (*n* = 2579) *n (%)*	Non-firearm Homicides (*n* = 3382) *n (%)*	Prevalence Difference (95% CI)
Sex				
*Male*	3833 (86.3)	1708 (88.7)	2125 (84.4)	4.3 (2.3, 6.3)
*Female*	610 (13.7)	217 (11.3)	393 (15.6)	−4.3 (−6.3, −2.3)
Race/Ethnicity				
*White, non-Hispanic*	2273 (46.1)	1105 (51.4)	1168 (42.0)	9.4 (6.6, 12.1)
*Black, non-Hispanic*	1138 (23.1)	471 (21.9)	667 (24.0)	−2.1 (−4.4, 0.3)
*Hispanic*	188 (3.8)	60 (2.8)	128 (4.6)	−1.8 (−2.9, 0.7)
*All other race*^*b*^	1329 (27.0)	514 (23.9)	815 (29.3)	−5.4 (−7.9, −3.0)
Age (years)				
*< 18*	122 (3.4)	54 (3.3)	68 (3.4)	−0.1 (−1.2, 1.1)
*18 to 59*	2611 (71.9)	934 (57.8)	1677 (83.2)	−25.4 (−28.3, − 22.4)
*60 and above*	898 (24.7)	627 (38.8)	271 (13.4)	25.4 (22.6, 28.2)
Perpetrator was caregiver for victim	236 (5.1)	86 (4.2)	150 (5.7)	−1.5 (−2.8, −0.3)
Perpetrator mentally ill	418 (13.8)	116 (8.7)	301 (17.7)	−8.9 (−11.3, −6.6)

Columns may not always equal to 100% due to rounding

^a^ Missing Data: Sex (1518 [25.5%]), Race/Ethnicity (1033 [17.3%]), Age (2330 [39.1%]), Perpetrator was caregiver of victim (1289 [21.6%]), Perpetrator mentally ill (2928 [49.1%])

^b^ all other race includes American Indian/Alaska Native (non-Hispanic), Asian/Pacific Islander (non-Hispanic), two or more races (non-Hispanic), other/unspecified (non-Hispanic)

Compared to non-firearm homicides (6%), firearm homicides (13%) more commonly involved multiple victims (prevalence difference = 7%; 95% CI: 5, 8%) (Table 3). Intimate partner violence was noted as the precipitating circumstance in 27% of firearm homicides vs. 9% of non-firearm homicides. The perpetrator died by suicide in 23% of the firearm homicides compared to 2% of non-firearm homicides (prevalence difference = 21%; 95% CI: 19, 22%). Of the 675 homicide-suicide incidents in the sample, 595 (88%) involved the use of a firearm. Mercy killing (e.g., victim was killed at the victim’s request, out of compassion to end their pain or distress) was considerably more common in firearm (3%) compared to non-firearm (0.3%) homicides.

**Table 3:** Distribution of older adult homicide incident characteristics using the National Violent Death Reporting System Data from 2003 to 2017

Incident Characteristics	Total (*N* = 5961) *N (%)*	Firearm Homicides (*n* = 2579) *n (%)*	Non-firearm Homicides (*n* = 3382) *n (%)*	Prevalence Difference (95% CI)
Homicide-suicide	675 (11.3)	595 (23.1)	80 (2.4)	20.7 (19.0, 22.4)
Multiple victim incident	551 (9.2)	338 (13.1)	213 (6.3)	6.8 (5.3, 8.3)
Legal intervention death of perpetrator	33 (0.6)	21 (0.8)	12 (0.4)	0.4 (0.06, 0.9)
Intimate partner violence	1003 (16.8)	687 (26.6)	316 (9.3)	17.3 (15.3, 19.3)
Precipitated by argument	1590 (26.7)	594 (23.0)	996 (29.5)	−6.5 (−8.7, −4.2)
Precipitated by another serious crime	1871 (31.4)	753 (29.2)	1118 (33.1)	−3.9 (−6.2, −1.5)
Mercy killing	79 (1.3)	70 (2.7)	9 (0.3)	2.4 (1.8, 3.1)
Drug involvement	240 (4.0)	100 (3.9)	140 (4.1)	−0.2 (−1.3, 0.7)
Led by Brawl	39 (0.7)	8 (0.3)	31 (0.9)	−0.6 (−1.0, − 0.2)
Gang-related	46 (0.8)	39 (1.5)	7 (0.2)	1.3 (0.8, 1.8)
Precipitated by Jealousy	50 (0.8)	32 (1.2)	18 (0.5)	0.7 (0.2, 1.2)
Justifiable self-defense	54 (0.9)	41 (1.6)	13 (0.4)	1.2 (0.7, 1.7)

From the narrative review, the majority of firearm incidents were in the context categories of (a) familial or intimate partner-related (37%), (b) Robbery or burglary (16%), (c) Altercation/dispute with non-family members (16%) and (d) Illness related (9%). Table 4 presents fictional examples of these contexts. Other contexts (6%) that appeared less frequently in the firearm narratives included police officers killed in the line of duty, mass shootings, serial killings, and victims caught between others firing. In 16% of the cases, not enough information was provided in the narrative to categorize the context. The narrative summaries from medical/coroner examiner and law enforcement almost always concurred on the context of the homicide in our random sample when both summaries were available for the cases.

**Table 4:** Examples of contexts of older adult homicides

Context	Definition	Example 1	Example 2
Familial or intimate partner-related	The homicide occurred in the context of intimate partner relationship issues or familial conflicts such as heated arguments, abuse, money, jealousy, cheating, divorce, and separation.	The perpetrator forcefully invaded the house and killed his wife and mother-in-law. The couple were separated. Victims’ relatives reported that he had threatened to kill the victim and her family previously.	A couple were arguing over financial expenses in the house. The argument escalated into a fight when the girlfriend shot her boyfriend with a firearm. The perpetrator fled and was arrested later from another area
Robbery or burglary	The homicide occurred in the context of a robbery or burglary by the perpetrator.	A group of three stopped a car on the highway. They robbed the victim and fled with the car after shooting the victim dead.	The victim was on the phone talking to his daughter. The perpetrator suddenly broke into the victim’s residence where he was living alone and asked for money. The daughter quickly reported to the police when she heard that an altercation started between the two followed by a gunshot sound. The police found the dead body of the victim on the floor.
Altercation/dispute with non-family members	The homicide was precipitated by an altercation or argument preceding the homicide. This excludes arguments with underlying relationship problems between intimate partners or other family members.	A man was shot and killed by his neighbor. On the day of the event, the victim and perpetrator reportedly had a verbal altercation. The two did not get along well according to their family members.	The victim died of remote complications from a gunshot wound that he sustained 10 years ago. Friends of the deceased informed that the victim was shot at a bar where he had a fight with some other guys trying to tease his girlfriend.
Illness related	The homicide was perpetrated to end the suffering of an ill victim or when the perpetrator was exhausted from taking care of the sick victim. Sometimes, both perpetrators and victims were sick, and, in some cases, only the perpetrator had an illness that precipitated the event.	A husband killed his wife who was suffering from terminal stage stomach cancer and then himself. He left a note that he was tired of looking after her for a long time and that he could no longer bear the sufferings of his partner.	The daughter was killed by her father using a firearm that the daughter owned. The old man was suffering from Alzheimer’s disease. The housemaid testified that the perpetrator was shouting at his daughter just before the incident for taking away the TV remote

Each example is a fictional scenario composed of multiple narratives and with intentional alterations

## Discussion

To our knowledge, this is the largest multi-state study to date examining victim, perpetrator, and incident characteristics of older adult homicides stratified by firearm status in the US. We used population-based data across 15 years in several states in addition to the Fatal Injury Data by WISQARS that allowed for a comprehensive determination of cases and rates over several years nationally allowing a better understanding of the trends in older adult homicides.

In our sample, firearms were the most common mechanism used in older adult homicides. This finding is consistent with the existing literature (Chu and Kraus 2004; Collins and Presnell 2006; Falzon and Davis 1998; Logan et al. 2019). Our trend examination revealed that firearm homicide rates in older adults have been steadily increasing since 2014 whereas non-firearm homicides in this population have relatively stable trends over time. A recent report suggested that in 2017, the US averaged over double the global homicide rate among all ages with the majority perpetrated by firearms (United Nations Office on Drugs and Crime 2019). In 2018, over a third of the US older adult population personally owned firearms (Smith and Son 2015). Previous literature has highlighted recent increases in firearm owning among older adults, which may contribute to a higher risk of homicide (Mertens and Sorenson 2012; Smith and Son 2015). A high growth rate of the older adult population coupled with an increasing trend in firearm homicide rates indicate an important need to develop and deploy effective prevention efforts.

We found that victims and perpetrators in both firearm and non-firearm homicides were mostly male. However, the gender difference was less pronounced for victims. The published literature supports this finding for overall homicides in older adult victims (Abrams et al. 2007). There are well-established associations between gender norms and violence perpetration. In particular, socially- and culturally-derived values and beliefs around conventional masculinity (e.g., men are expected to be strong and dominant) may contribute to perceptions of violence as an acceptable approach to resolving conflict, and thus a means of demonstrating adherence to conventional gender norms. The disproportionate impact of violence perpetration by males suggests maladaptive patterns of violent behavior that are maintained by dysfunctional adherence to particular versions of masculinity and male gender roles (Berke and Zeichner 2016; Levant et al. 2003).

The age and race distribution of victims and perpetrators in our study were also in line with prior findings (Rogers and Storey 2019). Older adults were typically killed in their own homes. The availability of firearms at home is thus a concern for older adult homicides as this exposure is known to increase homicide risks (Cummings et al. 1997; Dahlberg et al. 2004). Some older adults may choose to keep firearms in their homes for fear of being a crime victim due to their declining health conditions, especially if they lack social connections (Roberts and Willits 2013; Ross and Jang 2000). Self-protection has been identified as a motivating factor for carrying firearms among older adults (Smith 2001). Therefore, addressing fears of violence victimization directly may be an important component for preventing older adult homicides. Programs to promote awareness around unintended consequences of firearm availability in the household could be a possible strategy to test. This could also include dissemination and implementation of means safety interventions that are tailored for older adults.

We found differences in the victim-perpetrator relationship between firearm and non-firearm homicides. While most firearm homicides of older adults were perpetrated by intimate partners, non-firearm homicides were more often perpetrated by acquaintances and other family members. However, in both groups, family members and intimate partners in combination made up the largest group of perpetrators for older adult homicides than any other category of acquaintances including friends, colleagues, or strangers. Previous research with national incident-based data from Supplementary Homicide Reports from 1976 to 1985 indicated that homicide incidents among the population ages 65 or older were perpetrated more commonly by friends, acquaintances, or strangers compared to family members or intimate partners (Fox and Levin 1991). This was inconsistent with the present study and may indicate a shift in perpetrator-victim relationships since the 1980s. Our findings were consistent with more recent scholarship by Krienert & Walsh, which found that intimate partners tended to be the perpetrators of homicides involving females victims, while males were more likely to be killed by strangers (Krienert and Walsh 2010). We speculate that, as intimate partners are possibly more likely to have the knowledge about the presence of a firearm in the household and also about the storage details (e.g., storage location, locks), firearm homicides involved intimate partners as perpetrators more often than non-firearm homicides. Intimate partners of older adults may themselves be older adults and thus less physically strong, making a firearm an easier weapon to use than other mechanisms.

Nearly all homicide-suicide incidents involved the use of firearms in our study. This is consistent with prior literature, which also posits that homicide-suicides are distinct from homicide or suicide events (Saleva et al. 2007). In their article, Mertens & Sorenson presented that older adult homicide-suicide almost always involved intimate partners motivated by the prospect of ending the suffering of illness or another crisis. However, it could also be a result of intimate partner violence in this population (Malphurs and Cohen 2005; Mertens and Sorenson 2012). Studies have also highlighted the role of substance misuse, life stressors, and cultural norms around gender and mental health in these lethal acts (Mertens and Sorenson 2012). In this analysis, we found that intimate partner violence, jealousy, and mercy killing were more common among firearm than non-firearm homicides. The firearm incident narratives provided further insights into intimate partner homicides (i.e., intimate partner/familial and illness-related context). In our study, some perpetrators were caregivers of the older adults (who could be family members, intimate partners, or external caregivers). The existing literature indicates that older adults often suffer abuse and neglect by caregivers and family members (Acierno et al. 2010; Hunsaker 2014; Orfila et al. 2018) and are vulnerable to fatal consequences from those acts due to frailty and comorbidities. Karch and colleagues specifically examined the characteristics of homicides by caregivers. They found that 91% of the victims were aged 50 years or older, more than a third of the homicides were perpetrated with a firearm and a quarter of the incidents involved neglect (Karch and Nunn 2011). We recommend further investigations into homicide-suicide incidents among older adults to identify risk factors and inform interventions.

Our study provides valuable insights into the contexts of firearm homicides through the narrative reviews. While the predominant contexts for older adult homicides by firearms found in this study, such as illness, may not be unique to older adults but are likely more common among this population than the younger population. We expect such findings will help provide guidance to research and program efforts for the prevention of older adult homicides.

### Strengths and limitations

Our study’s strength lies in using recent and comprehensive data sources (Barber et al. 2013). Our focus on firearm vs. non-firearm comparison can guide preventive efforts and policy decisions. However, the findings of this study must be considered in light of some limitations. For some variables, there was notable missingness warranting caution in the interpretation of findings pertaining to them. Reported variables had missingness ranging from 17% (race/ethnicity) to 49% (mentally ill) for perpetrator characteristics and from 2% (marital status) to 21% (alcohol use when injured) for victim characteristics. Variables that involved information about victim in relation to the perpetrator were missing for over 30% of cases (32% for ‘prior history of victim abuse by perpetrator’ and 37% for ‘victim’s relationship with perpetrator’). It is important to note that, the incident circumstance variables in NVDRS data were coded as ‘no’ if abstractors were unable to confirm or did not know the information rather than a definitive absence of that circumstance. Moreover, completeness of the context categories presented is limited by the accuracy and depth of detail recorded in the medical examiner and law enforcement reports.

NVDRS data were available only for 36 states, Puerto Rico, and District of Columbia at the time of our analyses. Thus, the results might lack generalizability. Nonetheless, we observed that the victim characteristics of our sample were similar to those of the whole US population (supplemental Table S1). Future studies would benefit from examining more granular information on geographic location (e.g., rural/urban) for older adult homicide cases. Furthermore, since NVDRS data entirely consists of victims of violent deaths, we could not examine potential causes of increase in the rates of older adult homicides (Lyons et al. 2020). It would be valuable to understand why older adult homicide rates are increasing in a future study using other sources of data.

## Conclusion

Firearms are the most common mechanism used in older adult homicides. The overall and firearm-specific homicide rates in this population have been increasing since 2014. Most firearm-related homicides in older adults are perpetrated by current or former intimate partners. Multiple victim homicides are more common when firearms are involved. About one in four of firearm homicides involve subsequent suicide of the perpetrator. Further research to develop effective violence victimization prevention strategies among this group, especially those that limit access to firearms by potential perpetrators, is warranted.

## Supplementary Information

**Additional file 1.**

## Data Availability

The data that support the findings of this study are available from Centers for Disease Control and Prevention, but restrictions apply to the availability of these data, which were used under license for the current study, and so are not publicly available.

## Abbreviations

CDC: Centers for disease control and prevention
FBI: Federal bureau of investigation
NVDRS: National violent death reporting system
SHR: Supplementary homicide reports
WISQARS: Web-based injury statistics query and reporting system
