# Supplementary material for: Characteristics and patterns of older adult homicides in the United States
Source: Inj Epidemiol. 2021 Feb 1;8:5. doi: 10.1186/s40621-021-00299-w (PMC7849130; doi:10.1186/s40621-021-00299-w)
Supplement: Supplementary file 1 — Additional file 1. [file 40621_2021_299_MOESM1_ESM.docx]

**APPENDIX A**

Table S1: Comparison of older adult homicide victim characteristics between NVDRS and WISQARS during 2003 to 2017

|  | **NVDRS** | | | **WISQARS** | | |
| --- | --- | --- | --- | --- | --- | --- |
| **Victim Characteristics** | **Total**  (N= 6188)  *n (%)* | **Firearm Homicides** (N= 2710)  *n (%)* | **Non-firearm Homicides** (N= 3478)  *n (%)* | **Total**  (N=19561)  *n (%)* | **Firearm Homicides** (N=8394)  *n (%)* | **Non-firearm Homicides** (N=11167)  *n (%)* |
| Sex |  |  |  |  |  |  |
| *Male* | 3859 (62.4) | 1704 (62.9) | 2155 (62.0) | 12212 (62.4) | 5289 (63.0) | 6,923 (62.0) |
| *Female* | 2329 (37.6) | 1006 (37.1) | 1323 (38.0) | 7,349 (37.6) | 3105 (37.0) | 4,244 (38.0) |
| Race/Ethnicity |  |  |  |  |  |  |
| *White, non-Hispanic* | 4018 (64.9) | 1795 (66.2) | 2223 (63.9) | 12,605 (64.4) | 5,598 (66.7) | 7,007 (62.7) |
| *Black, non-Hispanic* | 1499 (24.2) | 668 (24.7) | 831 (23.9) | 4,473 (22.9) | 1,924 (22.9) | 2,549 (22.8) |
| *Hispanic* | 351 (5.6) | 122 (4.5) | 229 (6.6) | 1572 (8.0) | 563 (6.7) | 1009 (9.0) |
| *All other race^a^* | 320 (5.2) | 125 (4.6) | 195(5.6) | 911 (4.7) | 309 (3.7) | 602 (5.4) |
| Age (years) |  |  |  |  |  |  |
| *60 to 64* | 2123 (34.3) | 1026 (37.9) | 1097 (31.5) | 6,497 (33.2) | 3,190 (38.0) | 3,307 (29.6) |
| *65 to 74* | 2349 (38.0) | 1099 (40.6) | 1250 (35.9) | 7308 (37.4) | 3256 (38.8) | 4052 (36.3) |
| *75 to 84* | 1216 (19.7) | 459 (16.9) | 757 (21.8) | 4082 (20.9) | 1490 (17.8) | 2592 (23.2) |
| *85 and above* | 500 (8.1) | 126 (4.7) | 374 (10.8) | 1,674 (8.6) | 458 (5.5) | 1,216 (10.9) |

*^a^*In NVDRS all other race includes American Indian/Alaska Native (non-Hispanic), Asian/Pacific Islander (non-Hispanic), two or more races (non-Hispanic), other/unspecified (non-Hispanic). In WISQARS, all other race includes American Indian/Alaska Native (non-Hispanic) and Asian/Pacific Islander (non-Hispanic)

Table S2: Variable definitions from National Violent Death Reporting System used in the study

| **Variable** | **Definition** |
| --- | --- |
| Homicide | Deaths resulted from the intentional use of force or power against another person/s, irrespective of whether the intent was actual or only to threaten the victim |
| Homicide-suicide | A homicide-suicide incident was one where the perpetrator committed suicide within 24 hours of killing the victim/s the incident |
| Alcohol use suspected when injured | Victim suspected to use alcohol in the hours preceding the incident |
| Alcohol problem | Victim had alcohol dependence |
| Other substance problem | Victim had non-alcohol related substance abuse problem |
| Current diagnosed mental health problem | Victim had diagnosed mental health problem |
| Current depressed mood | Victim was reportedly perceived by self or others to be depressed at the time of injury |
| Current mental health or substance abuse treatment | Victim was under treatment for a mental health problem or substance abuse problem at the time of injury |
| Ever treated for mental health or substance abuse | History of the victim ever being treated for a mental health or substance abuse problem |
| Intimate partner violence | The homicide was related to immediate or ongoing conflict or violence between current or former intimate partners |
| Precipitated by argument | An argument of conflict led to the victim’s death |
| Precipitated by another serious crime | The murder was precipitated by another serious crime like drug dealing, robbery etc. |
| Mercy killing | Victim was killed, at the victim’s request, out of compassion in order to end his or her pain or distress |
| Drug involvement | Drug dealing, drug trade or drug use is suspected to have played a role in precipitating the incident |
| Led by Brawl | Immediately before the violent death, there was a mutual physical fight between three or more individuals which resulted in the death of individuals involved in the fight or, bystanders or individuals trying to stop the argument |
| Gang-related | Captures deaths that are classified as gang motivated (i.e. motive of the incident was gang related) or had suspected involvement of a gang member (i.e. gang member was a suspect or victim in the incident) |
| Precipitated by Jealousy | Jealousy or distress over a current or former intimate partner’s relationship or suspected relationship with another person led to the incident |
| Justifiable self-defense | The homicide was committed by a law enforcing officer in the line of duty or was committed by a civilian in legitimate self-defense or in defense of others |
| Victim was a bystander | The victim was a bystander and not the intended target |
| Victim was intervener assisting another victim | Victim was an intervener other than a law enforcement officer who was killed while assisting a crime victim |
| Victim used weapon | The victim used a weapon during the course of the incident |
| Prior history of victim abuse by perpetrator | physical, psychological, sexual or other abuse of the victim by the perpetrator confirmed or suspected |
| Perpetrator was caregiver for victim | Perpetrator was a caregiver for the victim |
| Perpetrator mentally ill | The perpetrator’s attack on the victim is believed to be the direct result of a mental illness |
| Victim was perpetrator of violence in past month | Victim was a perpetrator of violence within the past month that was distinct and occurred before the violence that killed the victim. |
| Victim experienced violence in past month | Victim experienced violence in the past month that was distinct and occurred before the violence that killed the victim |

Table S3: Distribution of variable missingness by mechanism of older adult homicides in NVDRS during 2003 to 2017

| **Variables with missing values** | **Firearm** | **Non-firearm** | **Full sample** |
| --- | --- | --- | --- |
|  | N (%) of missing | N (%) of missing | N (%) of missing |
| Victim variables |  |  |  |
| *Education level* | 245 (9.0) | 361 (10.4) | 606 (9.8) |
| *Marital status* | 43 (1.6) | 102 (2.9) | 145 (2.3) |
| *Victim’s relationship with perpetrator* | 1036 (38.2) | 1254 (36.1) | 2290 (37.0) |
| *Victim’s location when injured* | 83 (3.1) | 64 (1.8) | 147 (2.4) |
| *Served in military* | 178 (6.6) | 319 (9.2) | 497 (8.0) |
| *Homeless* | 113 (4.2) | 123 (3.5) | 236 (3.8) |
| *Alcohol use suspected when injured* | 677 (19.5) | 592 (21.6) | 1269 (20.5) |
| *Prior history of victim abuse by perpetrator* | 849 (31.3) | 1145 (32.9) | 1994 (32.2) |
| Perpetrator variables |  |  |  |
| *Sex* | 654 (25.4) | 864 (25.6) | 1518 (25.5) |
| *Race/Ethnicity* | 429 (16.6) | 604 (17.9) | 1033 (17.3) |
| *Age* | 964 (37.4) | 1366 (40.4) | 2330 (39.1) |
| *Perpetrator was*  *caregiver for victim* | 533 (20.7) | 756 (22.4) | 1289 (21.6) |
| *Perpetrator mentally ill* | 1249 (48.4) | 1679 (49.7) | 2928 (49.1) |
